# Depressive symptoms in Fabry disease: the importance of coping, subjective health perception and pain

**DOI:** 10.1186/s13023-020-1307-y

**Published:** 2020-01-28

**Authors:** Simon Körver, Gert J. Geurtsen, Carla E. M. Hollak, Ivo N. van Schaik, Maria G. F. Longo, Marjana R. Lima, Leonardo Vedolin, Marcel G. W. Dijkgraaf, Mirjam Langeveld

**Affiliations:** 1 Department of Endocrinology and Metabolism, Amsterdam UMC, location AMC, University of Amsterdam, Meibergdreef 9, Amsterdam, The Netherlands; 2 Department of Medical Psychology, Amsterdam UMC, location AMC, University of Amsterdam, Meibergdreef 9, Amsterdam, The Netherlands; 3 Department of Neurology, Amsterdam UMC, location AMC, University of Amsterdam, Meibergdreef 9, Amsterdam, Spaarne Gasthuis, Haarlem The Netherlands; 4 Department of Radiology, Massachusetts General Hospital, Boston, MA USA; 5 Department of Radiology, Hospital Moinhos de Vento, Porto Alegre, Brazil; 6 Imaging Director, Diagnóstico da América SA, Sao Paulo, Brazil; 7 Department of Clinical Epidemiology, Biostatistics and Bioinformatics, Amsterdam UMC, location AMC, University of Amsterdam, Meibergdreef 9, Amsterdam, The Netherlands

**Keywords:** Fabry disease, Depressive symptoms, Depression, Coping, Pain, Health perception

## Abstract

**Background:**

Despite the high prevalence of depressive symptoms in Fabry disease (FD), it is unclear which patient characteristics are important in relation to these symptoms. Additionally, the impact of coping styles in relation to depressive symptoms in FD has been unexplored. Determining the impact of different factors relating to depressive symptoms in FD can guide both prevention and treatment of these symptoms.

**Methods:**

Depressive symptoms (Center for Epidemiologic Studies Depression scale (CESD)) and coping styles (Utrecht Coping List) were assessed in a Dutch FD cohort. Other potentially important variables were identified from FD literature and assessed in this cohort. Relations were evaluated using multiple linear models.

**Results:**

Potentially important variables in FD literature were: pain, unemployment, health perception, being single, comorbidities and stroke. Employed coping styles were “avoidance and brooding”, “positivity and problem solving” and “seeking social support”. Thirty-one of the 81 FD patients (38%) had depressive symptoms. CESD-scores were lower in patients with better health perception and more “positivity and problem solving” and higher in patients with more pain and “avoidance and brooding”. The best model explained 70% (95%CI: 54–76%) of observed variance of the CESD.

**Conclusions:**

Depressive symptoms in FD are related to pain, negative health perception and use of specific coping styles. Psychological interventions could be employed to alter coping behavior and alleviate depressive symptoms.

## Background

Fabry disease (FD; OMIM 301500) is a rare X-inherited lysosomal storage disorder. Accumulation of globotriaosylceramide and related compounds occurs in various cell types due to deficiency of α-galactosidase A activity (enzyme commission no. 3.2.1.22). Accumulation of those substrates may result in damage of the kidneys, heart and brain [1]. Important predictors of symptoms and complications in FD are sex and phenotype [2]. Generally, men have more and earlier complications and are more severely affected compared to women. In addition, patients with a classical FD phenotype are often more severely affected compared to patients with a non-classical FD phenotype [2]. A high prevalence of depressive symptoms (46%) has been reported in patients with FD compared to the general population [3–6]. FD related factors, such as pain [3, 4] and non-FD related factors such as being single [4] or lack of social support [7] have been related to depressive symptoms in FD in earlier studies. It has been hypothesized that the cerebral pathology in FD might be a biological substrate for depressive symptoms [3, 6, 7]. Interestingly, while most studies failed to establish a relation between organ involvement and depressive symptoms [5, 8], FD patients’ perception of their health was strongly related to depressive symptom severity [4, 9]. This relationship is not unique for FD and has been shown in other diseases as well [10]. While many patients living with a chronic disease show resilience and manage to adapt to new situations, such adjustment is hampered in a substantial subgroup [11]. Coping, a process of cognitive and behavioral effort to manage daily hassles as well as stressors that tax or exceed the resources of a person [12], might be an important factor in the psychological adjustment to a chronic disease like FD. In chronic diseases such as rheumatoid arthritis or type 2 diabetes, different coping styles have been related to both improvement and worsening of psychological [13] and physical outcomes [13, 14].

Determining the importance of different factors in relation to depressive symptoms in FD can support the identification of patients at risk as well as a starting point for FD specific (psychologic) interventions to prevent or treat depressive symptoms. Previous studies explored different variables in relation to depressive symptoms making it difficult to determine which factors should receive more attention and which can be ignored. Moreover, coping styles have not been previously assessed in relation to depressive symptoms in patients with FD. The purpose of this study was therefore: 1) To identify potentially important variables related to depressive symptoms in FD through a literature search and to evaluate the effect of these in our patient cohort; 2) To evaluate coping styles in relation to depressive symptoms in FD; 3) To explore further potential variables of interest in relation depressive symptoms in FD.

## Methods

### Study design and data collection

**Fig. 1:**
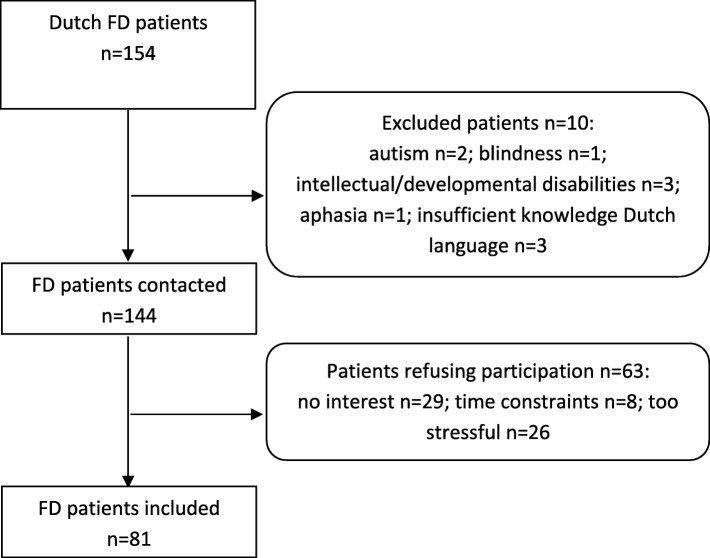
Flow chart of non-participants and in- and excluded patients. FD = Fabry disease

The Amsterdam University Medical Center (location Academic Medical Center (AMC)) is the national referral center for FD. Adult Fabry patients (*n* = 154) at the AMC were screened for eligibility (Fig. 1). All included patients filled out questionnaires and completed a comprehensive neuropsychological assessment, between July 2016 and April 2017. The tests were performed at the AMC outpatient clinic or during a home visit. The neuropsychological data have been published elsewhere [15]. Demographic, clinical and disease characteristics were extracted from a local clinical database and cross-checked with medical records. Patients were phenotypically characterized as having classical or non-classical FD using established criteria [15, 16].

### Identification of variables related to depressive symptoms in FD

Studies were identified using: 1) a systematic review giving an overview of studies on depressive symptoms in FD until November 2012 [3] and 2) a PubMed search until the 7th of January 2019. We used an extended version of the search from the systematic review [3] including synonyms of “Fabry disease”, “depression” and “psychology” (see Additional file 3: Supplemental identified variables: Search for studies). Variables were extracted and classified as “related to depressive symptoms in FD” or as “unrelated to depressive symptoms in FD”.

### Depressive symptoms

The Center for Epidemiologic Studies Depression scale (CESD) was used to quantify depressive symptoms [17]. Twenty items are scored on a four point Likert scale (range 0 to 3) resulting in a score between 0 and 60. Scores ≥16 indicate the presence of depressive symptoms [4, 17].

### Coping

Coping was measured using the Utrecht Coping List (UCL, a Dutch version of the Coping Scale by Westbrook [18]), a questionnaire consisting of 47 items measuring seven coping styles (palliative, passive, active, avoiding, social support seeking, reassuring thoughts, expressing emotions) [19]. Responses are rated on a scale ranging from 1 (seldom or never) to 4 (very often) and can be added to a total score per coping style, with higher scores indicating stronger use of that coping style. Coping is regarded as a personality style, meaning that most people have a regular way of coping with stressors but might change this style somewhat depending on the situation [19].

### Neuropsychological test battery and subjective cognitive complaints

All included patients completed 16 well-established neuropsychological tests assessing: language, memory, visuospatial perception, processing speed and executive functioning (for specific neuropsychological tests see [15]). Presence or absence of objective cognitive impairment was determined using preset criteria (see Additional file 1: Supplemental methodology: Objective cognitive impairment). Subjective cognitive complaints were assessed in a structured interview and rated as present or absent.

### Additional questionnaires

Pain was assessed using the Brief Pain Inventory (BPI) with scores graded from 0 (absence of pain) to 10 (worst possible pain) [20]. For this study we used the BPI severity score. This score is an average of four items: worst pain, least pain, average pain and pain right now [21].

The 36-item short form survey (SF-36) is a health related quality of life (QoL) questionnaire, consisting of 36 items. The SF-36 assesses eight domains of QoL on a scale from 0 to 100, with higher scores indicating better functioning [22]. In this study we focused on the following subscales: subjective health perception, fatigue and self-rated social functioning.

Sleep quality was measured using the Pittsburgh Sleep Quality Index (PSQI) [23]. Total scores range from 0 to 21 and a score > 5 is indicative of poor sleep quality.

### Clinical characteristics, complications and comorbidities

We calculated left ventricular mass, rated cardiac fibrosis and calculated estimated glomerular filtration rate (see Additional file 1: Supplemental methodology: Clinical characteristics and complications for additional information). Stroke was diagnosed by a neurologist using a combination of clinical symptoms and MRI (if available). Comorbidity was defined as presence or absence of an additional (chronic) somatic disorder.

### Brain MRI

Routine follow-up scans were performed on a yearly or biannual basis using a 3T system (Philips Ingenia, Philips Medical Systems, Best, The Netherlands), using a standardized protocol [15]. Two neuroradiologists rated the MRIs, (*MRL* evaluated basilar artery pathology, *MGL* evaluated infarctions and white matter lesions (WMLs)), blinded for all patient characteristics. WMLs were rated on axial FLAIR using the Fazekas scale, ranging from 0 (no WMLs) to 6 (confluent periventricular and deep WMLs) [24].

### Statistical methods

R (version 3.5.1) was used for statistical analysis. *P*-values < 0.05 were regarded as significant, unless stated otherwise.

Firstly, an exploratory factor analysis (EFA) was performed on the UCL. The rationale was that adding the original seven subscales of the UCL to a multiple regression analysis would complicate adding other variables due to limited power. The EFA reduced the number of UCL scales for the multiple regression analysis while providing a reflection of coping styles employed by FD patients.

The EFA in short: We adjusted the EFA-methodology for the non-normality and ordinal nature of the data. Factors were named using the items with the strongest loading per factor and factor scores were calculated according to the Anderson-Rubin method [25]. This results in continuous scores with a mean of 0 and a change in factor score of 1 per SD increase or decrease. Most scores will range between − 2 to 2, and higher scores indicate more extensive use of the coping style in question.

UCL factor scores were split by sex and phenotype. A one-way ANOVA with Bonferroni correction was performed to compare factor scores.

Secondly, two multiple linear regression models were created with CESD score as the outcome variable. Model 1 was used to evaluate the effect of important variables identified in previously published FD research i.e. previously significantly related to depressive symptoms in FD and available in our cohort. In Model 2 we extended Model 1 with the coping styles identified using EFA. Assumptions of both models were assessed and we performed sensitivity analyses removing outliers/influential patients to test the robustness of the findings.

Lastly, we explored the effects of other potentially interesting variables in relation to depressive symptoms in FD, using an akaike information criterion based explorative automated model generating procedure. The explorative automated procedure specified all possible models with the given set of variables and presents model-averaged importance of variables (See Additional file 1: Supplemental methodology: statistical methods for additional information on abovementioned analyses). In the explorative automated model generating procedure we added the variables of Model 2 as well as variables that are important in depression research in the general population but seemed less important or have never been explored in previous FD literature.

Results were reported in accordance with the Strengthening the Reporting of Observational studies in Epidemiology guidelines [26].

## Results

### Patients

There were no significant differences between participants and excluded patients/non-participants in sex, phenotype, age, history of stroke or median Fazekas score [15]. A total of 81 patients were included, 52.6% of the Dutch Fabry cohort (Fig. 1), with a mean age of 44.5 ± 14.3 years (range: 19–76 years) (Table 1). Twenty-eight patients were men (34.6%), 60 patients (74.1%) had a classical phenotype and 43 patients (53.1%) were currently treated with enzyme replacement therapy. Twenty-two patients (27.2%) reported a history of, or current, depression. WML severity was generally mild, but in some patients with classical disease Fazekas scores ranged up to 6, indicating presence of severe confluent WMLs.

**Table 1:** Patient characteristics

	All	Men		Women	
		Classical	Non-classical	Classical	Non-classical
Patients, n (%)	81	17 (21.0%)	11 (13.6%)	43 (53.1%)	10 (12.3%)
Age in years, mean (±SD)	44.5 (±14.3)	38.6 (±13.5)	58.0 (±11.2)	43.5 (±13.9)	43.9 (±13.0)
Currently on ERT, n (%)	43 (53.1%)	15 (88.2%)	2 (18.2%)	25 (58.1%)	1 (10.0%)
Years treated with ERT, median (range)	1.6 (0.0–16.0)	12.4 (1.5–16.0)	0.0 (0.0–14.2)	1.6 (0.0–13.6)	0.0 (0.0–0.3)
Unemployed^a^, n (%)	32 (39.5%)	9 (52.9%)	5 (45.5%)	15 (34.9%)	3 (30.0%)
Unfit for work^b^, n (%)	20 (24.7%)	7 (41.2%)	2 (18.1%)	10 (23.3%)	1 (10.0%)
Single^c^, n (%)	30 (37.0%)	9 (52.9%)	4 (36.4%)	14 (32.6%)	3 (30.0%)
Years of education, mean (±SD)	13.8 ± 3.0	14.4 ± 2.8	13.9 ± 4.9	13.3 ± 2.7	14.9 ± 1.8
Depression^d^, n (%)	22 (27.2%)	3 (17.6%)	3 (27.3%)	12 (27.9%)	4 (40.0%)
Burnout^d^, n (%)	12 (14.8%)	1 (5.9%)	0 (0.0%)	7 (16.3%)	4 (40.0%)
Current psychiatric medication, n (%)	15 (18.5%)	2 (11.8%)	3 (27.3%)	9 (20.9%)	1 (10.0%)
Antidepressants, n (%)	7 (8.6%)	1 (5.9%)	2 (18.2%)	3 (7.0%)	1 (10.0%)
Benzodiazepines, n (%)	9 (11.1%)	1 (5.9%)	1 (9.1%)	7 (16.3%)	0 (0.0%)
Loneliness, n (%)	11 (13.6%)	2 (11.8%)	2 (18.2%)	6 (14.0%)	1 (10.0%)
Comorbidity, n (%)	40 (49.4%)	8 (47.1%)	10 (90.9%)	19 (44.2%)	3 (30.0%)
Left ventricular hypertrophy^e,f^, n (%)	45 (55.6%)	13 (76.5%)	4 (36.4%)	24 (55.8%)	4 (40.0%)
Cardiac fibrosis, n (%)	23/72 (31.9%)	6/17 (35.3%)	2/6 (33.3%)	14/39 (35.9%)	1/10 (10.0%)
eGFR< 60 ml/min, n (%)	11 (13.6%)	2 (11.8%)	4 (36.4%)	5 (11.6%)	0 (0.0%)
Fazekas score^e,g^, median (range)	1 (0–6)	0 (0–6)	1 (0–3)	1 (0–6)	0.5 (0–2)
Complications, n (%)	27 (33.3%)	7 (41.2%)	6 (54.5%)	14 (32.6%)	0 (0.0%)
Cardiac, n (%)	14 (17.3%)	4 (23.5%)	4 (36.4%)	6 (14.0%)	0 (0.0%)
Renal, n (%)	4 (4.9%)	1 (5.9%)	2 (18.2%)	1 (2.3%)	0 (0.0%)
Stroke, n (%)	10 (12.3%)	2 (11.8%)	2 (18.2%)	6 (14.0%)	0 (0.0%)

Continuous variables are presented as median (range) or mean (±SD) and discrete variables as number (percentages)

*ERT* enzyme replacement therapy, *eGFR* estimated glomerular filtration rate

^a^Includes three retirees

^b^Includes three patients regarded partially unfit for work

^c^Unmarried, divorced or widowed

^d^History of or current, as diagnosed by a general practitioner, psychologist or psychiatrist

^e^MRIs were unavailable in seven patients (three non-classical men, four classical women) due to presence of an MRI non-compatible pacemaker or ICD (*n* = 6) and due to claustrophobia (*n* = 1)

^f^If MRI of the heart was not available then presence of left ventricular hypertrophy on echocardiography was used

^g^In one patient the brain MRI was performed in a different hospital

### Depressive symptoms and neuropsychological functioning

A total of 31 patients (38.3%) experienced depressive symptoms (score of ≥16 on the CESD) and scores ranged from 0 to 44 (Table 2). The presence of depressive symptoms was evenly spread over subgroups defined by sex and phenotype. Thirteen patients (16.0%) were classified as having objective cognitive impairment, of which seven were men with classical FD (41.0%) and none were women with non-classical FD.

**Table 2:** Questionnaires, scales and cognition

	All	Men		Women	
		Classical	Non-classical	Classical	Non-classical
CESD, median (range)	11 (0–44)	11 (0–40)	12 (0–37)	12 (0–44)	7.5 (0–20)
CESD≥16, n (%)	31 (38.3%)	7 (41.2%)	4 (36.4%)	17 (39.5%)	3 (30.0%)
Subjective cognitive complaints^a^, n (%)	52 (64.2%)	11 (64.7%)	5 (45.5%)	31 (72.1%)	5 (50.0%)
Objective cognitive impairment^b^, n (%)	13 (16.0%)	7 (41.0%)	3 (27.3%)	3 (7.0%)	0 (0%)
BPI severity, median (range)	1.0 (0.0–7.0)	0.8 (0.0–6.5)	4.0 (0.0–7.0)	2.0 (0.0–7.0)	0.0 (0.0–5.8)
PSQI, median (range)	5.0 (0.0–20.0)	4.0 (0.0–14.0)	6.0 (1.0–13.0)	6.0 (1.0–20.0)	5.5 (2.0–10.0)
PSQI> 5, n (%)	39 (48.1%)	4 (23.5%)	7 (63.6%)	23 (53.5%)	5 (50.0%)
SF-36 Fatigue, mean (±SD)	50.5 (±23.0)	55.3 (±24.8)	54.5 (±22.2)	45.5 (±22.0)	59.5 (±22.4)
SF-36 Social functioning, mean (±SD)	71.5 (±26.8)	75.7 (±24.8)	69.3 (±28.2)	67.4 (±28.4)	83.8 (±18.7)
SF-36 Health perception, mean (±SD)	43.3 (±22.6)	41.2 (±25.0)	40.0 (±23.2)	40.5 (±19.1)	63.0 (±25.3)

Continuous variables are presented as median (range) or mean (±SD) and discrete variables as number (percentages)

*CESD* Center for Epidemiologic Studies Depression scale, *BPI* Brief Pain Inventory, *PSQI* Pittsburgh Sleep Quality Index, *SF-36* Short Form-36 Health Survey

^a^Presence or absence of subjective cognitive complaints

^b^presence or absence of objective cognitive impairment

### Exploratory factor analysis of the Utrecht coping list

EFA of the UCL data resulted in three coping styles. These styles will be referred to as *“avoidance and brooding”*, *“positivity and problem solving”* and *“seeking social support and comfort”*. See additional file 2: Supplemental results: EFA for further information.

There were no significant differences in employment of coping styles between the FD subgroups divided by sex and phenotype (avoidance and brooding: F(3,77) = 0.28, *p* = 0.84; positivity and problem solving: F(3,77) = 0.87, *p* = 0.46; social support and comfort: F(3,77) = 2.42, *p* = 0.07).

### Identified variables and multiple linear regression

A total of 16 studies assessed the relation between one or more variables and depressive symptoms in FD (see Additional file 3: Supplemental identified variables: Depressive symptoms in FD literature and variables of interest for details). Six variables were found to be significantly related to depressive symptoms in earlier FD studies (i.e. BPI severity score, being unfit for work, SF-36 health perception score, being single, presence of comorbidities and history of stroke). These variables were added in Model 1. Model 1 explained 43.3% of CESD score variance (F(6,74) = 9.43, *p* < 0.0001, 95%CI 24.3–53.7%, adjusted R^2^ 39.3%) (Table 3). CESD scores were positively related to higher BPI severity scores and negatively related to higher SF-36 health perception and to presence of a comorbidity.

**Table 3:** Summary of multiple linear regression Model 1 and 2

Model 1					Model 2			
Independent variables	*B* (95% CI)	SE *B*	β	p-value	*B* (95% CI)	SE *B*	β	p-value
BPI severity	1.60 (0.63–2.58)	0.49	0.35	0.002	0.82 (0.04–1.59)	0.39	0.18	0.039
Unfit for work	0.23 (−5.22–5.69)	2.74		0.933	0.47 (−3.57–4.51)	2.03		0.817
SF-36 Health perception	−0.19 (−0.30 – −0.09)	0.05	− 0.41	< 0.001	− 0.13 (− 0.21 – − 0.05)	0.04	−0.28	0.001
Single	−0.49 (−4.42–3.43)	1.97		0.804	− 0.60 (− 3.55–2.35)	1.48		0.687
Comorbidity	−6.15 (−10.20 – −2.10)	2.03		0.003	−2.92 (− 6.07–0.23)	1.58		0.069
Stroke	3.18 (− 3.02–9.39)	3.11		0.309	3.18 (− 1.41–7.77)	2.30		0.171
Avoidance and brooding					5.39 (3.82–6.95)	0.79	0.50	< 0.0001
Positivity and problem solving					−3.12 (−4.53 – −1.71)	0.71	−0.29	< 0.0001
Seeking social support and comfort					−0.14 (−1.56–1.29)	0.72	−0.01	0.849
Intercept	20.74				18.14			
F-value	9.43			< 0.0001	18.68			< 0.0001
R^2^	43.3% (24.3–53.7)				70.3% (53.9–75.9)			
Adjusted R^2^	39.3%				67.1%			

*B* beta coefficients, *β* standardized beta coefficients for continuous variables, *SE* standard Error, *BPI* Brief Pain Inventory, *SF-36* Short Form-36 Health Survey

Model 2 investigated the coping styles identified with EFA in relation to the CESD scores (Avoidance and brooding, positivity and problem solving, seeking social support and comfort), in addition to the six variables from Model 1. Model 2 explained 70.3% of CESD score variance (F(9,71) = 18.68, *p* < 0.0001, 95%CI 53.9–75.9%, adjusted R^2^ 67.1%) (Table 3). CESD scores in this model were positively related to higher BPI severity scores and to higher avoidance and brooding. CESD scores were negatively related to higher SF-36 health perception and to more employment of positivity and problem-solving. The avoidance and brooding coping style had the greatest effect on CESD scores considering the standardized beta coefficients.

Overall, assumptions of both linear models were met. Sensitivity analyses, removing outliers and patients with most influence on the models fit, revealed no major differences in the model results (Additional file 2: Supplemental results: Assumption testing).

### Explorative automated model generation

**Fig. 2:**
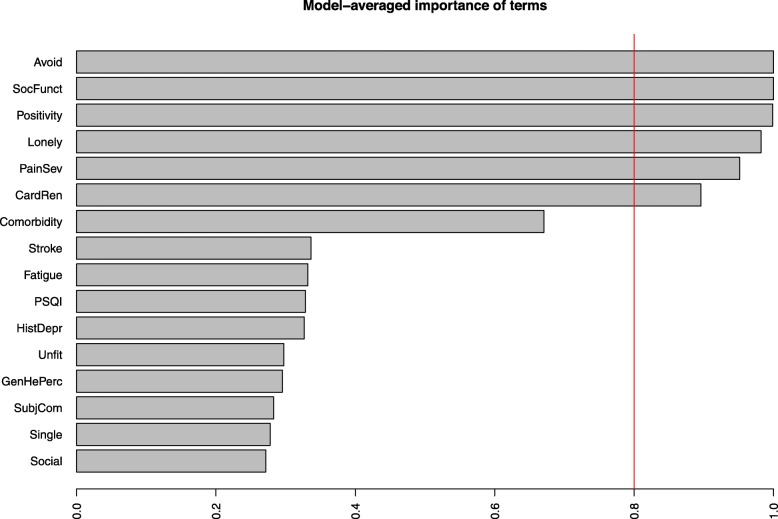
Results explorative models. Model averaged importance of the 6500 models explaining most variance of CESD-scores. Avoidance and brooding and SF-36 social functioning were included in all 6500 models and therefore set to 1.0. All variables with a model averaged importance > 0.8 might be relevant variables in relation to the CESD score. Avoid = Avoidance and brooding, SocFun = Short Form-36 Health Survey (SF-36) social functioning, Positivity = Positivity and problem solving, Lonely = Loneliness, PainSev = Brief pain inventory severity, CardRen = Cardiac and/or renal involvement, Fatigue = SF-36 fatigue, PSQI = Pittsburgh sleep quality index, HistDepr = History of depression, Unfit = unfit for work, GenHePerc = SF-36 general health perception, SubjCom = Subjective cognitive complaints, Social = Social support and comfort

Another seven variables of interest were identified and included in the automated explorative models with the CESD score as outcome variable (Additional file 3: Supplemental identified variables: Variables related to depressive symptoms in the general population). Added to all variables from Model 2 were: presence of loneliness, cardiac and/or renal involvement, SF-36 fatigue scale, self-rated sleep quality (PSQI), history of depression, subjective cognitive complaints and SF-36 self-rated social functioning scale. Of all these variables the avoidance and brooding and the positivity and problem solving coping styles, the SF-36 social functioning scale, presence of loneliness, the BPI severity score and cardiac and/or renal involvement explained the most CESD variance (Fig. 2).

### Post hoc analyses

Model 1 and 2 showed that presence of a comorbidity was negatively related to the CESD score, mainly in Model 1. Hypertension and hypercholesterolemia were the most prevalent comorbidity noted in our cohort (~ 50% of patients with a comorbidity), which were well regulated and not leading to symptoms. The negative relation between comorbidities and the CESD score decreased when Model 1 was adjusted by excluding hypertension and hypercholesterolemia (*B* − 3.58; *p* = 0.17; 95%CI -8.71 – 1.55).

Since the Fazekas score was not available for all patients we did not incorporate it in the explorative models. A linear model showed no relation between the Fazekas score and the CESD score (one-point increase: *B* 0.61; *p* = 0.43; 95%CI -0.92 – 2.13). There was no relation between presence of objective cognitive impairment and the CESD score or between sex and phenotype and the CESD score [15]. Lastly, we found no relation between years treated with enzyme replacement therapy and the CESD score (one treatment year increase: *B* 0.06; *p* = 0.79; 95%CI -0.37 – 0.48).

## Discussion

In this cross-sectional cohort study including more than half of the Dutch FD patients we found a high prevalence of depressive symptoms (38%), comparable to earlier work in FD patients [4]. We determined the importance of coping, in addition to variables identified from FD literature in relation to depressive symptoms in FD. An avoidant and brooding coping style was related to a higher depressive symptom score, while more a positive and problem-solving coping style was related to a lower score. Pain and a negative health perception, variables identified from FD literature, were also independently related to depressive symptoms. Of interest, while previous studies suggested a relation between unemployment and depressive symptoms, this was not confirmed in our model. Years treated with enzyme replacement therapy showed no relation to depressive symptoms. By using exploratory analyses we identified loneliness, experienced social functioning and cardiac/renal involvement as potentially important factors, which merit further research.

While this study is the first to explore coping in relation to depressive symptoms in FD, similar relations between coping styles and depressive symptoms were found in more common chronic diseases such as type 2 diabetes and rheumatoid arthritis. Avoidance [13] and brooding [27] are generally considered maladaptive and have been related to a higher prevalence of depressive symptoms in these diseases [13, 14, 27]. A positive mentality has been consistently related to lower rates of depressive symptoms [28]. In addition, problem-solving interventions have been effectively employed to decrease depressive symptoms in the general population [29]. The underlying assumption for these interventions is that for rational problem solving, a positive problem orientation is indispensable [29]. While FD itself is not directly “solvable” for the patients, research has shown that a problem-solving approach of intermediate goals (e.g. lifestyle adjustments, scheduling hospital appointments) improves self-management in for example type 2 diabetes patients [30].

The relation between social support and depressive symptoms has been less clear in chronic disease research. In early theoretical work, it was expected that seeking social support was related to better psychological outcomes [13]. However, we found no relation between seeking social support and depressive symptoms in the FD cohort. An explanation might be that chronic disease can complicate social support due to prolonged strain on the caregiver [31]. It has therefore been postulated that *seeking* social support is not similar to *receiving* social support and that social support might decrease during a prolonged disease course [13, 31]. In line with this, our explorative analyses showed that both subjective social impairment and loneliness may contribute to depressive symptoms in FD, meaning that expecting social support, but receiving less then desired, might increase depressive symptoms.

While we did not assess the relation between pain and coping in this study, the interrelation between pain, coping and depressive symptoms is likely complex [32]. Coping styles probably influence pain experience and the effect of treatment on pain in FD [3]. Moreover, a study testing a psychological counseling intervention for depressive symptoms in FD patients showed that pain seems to improve when depressive symptoms decrease [33]. It is also likely that depressive symptoms will improve with adequate treatment of pain.

Interestingly, while subjective health perception has been repeatedly identified as an important factor in relation to depressive symptoms in FD, the observed relation between organ complications and depressive symptoms has been less straightforward. We propose that impact of FD on patients’ perceived health extends beyond the physical symptoms and complications, to more subjective factors such as uncertainty about the future, difficulties surrounding heritability and stigmatization [34, 35]. In other words, complications and symptoms might have an effect on depressive symptoms, but the perception that patients have of their disease and the extent to which certain coping styles are employed will determine the individual outcome.

Of note, we could not confirm the previously observed relation [8] between a history of stroke and depressive symptoms, nor was there a relation between WMLs and depressive symptoms. This further strengthens the hypothesis that brain abnormalities are not the main cause of depressive symptoms in patients with FD [3, 5].

This study has several limitations. Although the sample size is large for a rare disease like FD, it limited our statistical analyses. Our multiple linear regression models are probably not adequate to detect small to medium effects, and results should be interpreted as such. Moreover, although background characteristics of included patients and non-participants were similar, there might be an inclusion bias: patients with more depressive symptoms might have had greater interest in participation. Conversely, severely depressed patients might have felt unable to participate due to depression related symptoms. Furthermore, we did not find a relation between years treated with enzyme replacement therapy and depressive symptoms. This analysis might be affected by indication bias: more severely affected patients are probably treated earlier and longer. This hampers strong conclusions on the effectiveness of enzyme replacement therapy on depressive symptoms. Lastly, we used an explorative automated model selection procedure. Since this automatically tested > 65,000 models this presents an extreme case of multiple testing, which warrants confirmation.

Future studies could further unravel the interrelation between pain, coping and depressive symptoms in FD patients by evaluating the mediating effect of coping between pain and depressive symptoms. Moreover, factors that influence patients’ health perceptions (e.g. illness perception, repeated medical testing) could be explored. Lastly, an extension to children and adolescents would be valuable, since coping strategies differ per life stage, as do FD related symptoms.

Finally, we recommend that pain, should be routinely assessed, monitored and treated according to published guidelines [36]. Considering the probable under-diagnosis and under-treatment of depressive symptoms in FD [4, 6] we further recommend to include a screening questionnaire (for example the CESD or the Beck Depression Inventory) in routine clinical care [37]. Patients with depressive symptoms should be referred, preferentially to psychologists with knowledge of chronic diseases [37].

## Conclusions

Depressive symptoms are frequent in patients with FD and are related to pain, negative health perception and use of specific coping styles. Future psychological treatment can be tailored to coping styles, for example by focusing on improvement of problem solving or decreasing avoidant behavior, ideally in a research setting.

## Supplementary information

**Additional file 1.** Supplemental methodology

**Additional file 2.** Supplemental results

**Additional file 3.** Supplemental identified variables

## Data Availability

The data sets generated and analyzed during the current study are not publicly available. Because of the rarity of the disease, even anonymized can be linked to a specific individual. In case of a specific scientific question, requests to make part of the data set available will be reviewed.

## Abbreviations

AMC: Academic Medical Center
BPI: Brief Pain Inventory
CESD: Center for Epidemiological Studies Depression Scale
EFA: exploratory factor analysis
FD: Fabry disease
PSQI: Pittsburgh Sleep Quality Index
QoL: quality of life
SF-36: 36-item Short Form Survey
UCL: Utrecht Coping List
WMLs: white matter lesions
