# Supplementary material for: Depressive symptoms in Fabry disease: the importance of coping, subjective health perception and pain
Source: Orphanet J Rare Dis. 2020 Jan 28;15:28. doi: 10.1186/s13023-020-1307-y (PMC6986064; doi:10.1186/s13023-020-1307-y)
Supplement: Supplementary file 1 — Additional file 1. Supplemental methodology [file 13023_2020_1307_MOESM1_ESM.docx]

**Supplemental methodology**

*Contents*

- Objective cognitive impairment
- Clinical characteristics
- Complications
- Statistical methods
  - Exploratory factor analysis (EFA) methodology
  - Assumption testing multiple linear regression models
  - Calculated statistics multiple linear models
  - Exploratory models

*Objective cognitive impairment*
Objective cognitive impairment (OCI) was defined as a T-score ≤33 on two or more distinct cognitive tests (T-scores ≤33 imply scoring <5^th^ percentile or 1.67 SD below the mean T-score of the normative population of 50). To decrease family-wise error rate two or more T-scores ≤33 on cognitive tests assessing a similar cognitive process were treated as a single deficient test score.

*Clinical characteristics*Kidney function was assessed using the estimated glomerular filtration rate (eGFR in ml/min/1.73m^2^), calculated using the CKD-EPI formula (1). Left ventricular mass on MRI was calculated without papillary muscles and adjusted for body surface area (Dubois formula). If no MRI was available left ventricular mass was calculated using echocardiography (Cube formula), adjusted for height^2.7^ (2). Presence of left ventricular hypertrophy was defined as >72 g/m^2^ or >49 g/m^2.7^ in men and >55 g/m^2^ or >47 g/m^2.7^ in women on MRI and echocardiography, respectively (3, 4). Late gadolinium enhancement on cardiac MRI was regarded indicative of the presence of fibrosis.
For our explorative automated model selection procedure (see statistical methods) we created an ordinal scale rating severity of cardiac and renal involvement from 0-2: (0) No renal or cardiac involvement, (1) renal involvement (eGFR <60 ml/min) and/or cardiac involvement (left ventricular hypertrophy and/or fibrosis), (2) cardiac and/or renal complications.

*Complications*An eGFR <15 ml/min/1.73m^2^ (1), a history of renal transplantation and/or a history of dialysis were regarded as renal complications. A history of myocardial infarction, coronary artery bypass grafting, percutaneous transluminal coronary angiography, hospitalization due to heart failure, arrhythmias (including atrial fibrillation) and presence of a pacemaker or ICD were regarded as cardiac complications.

*Statistical methods
Exploratory factor analysis (EFA) methodology*An exploratory factor analysis (EFA) was performed on the Utrecht Coping List (package: psych (5)). We used the Kaiser-Meyer-Olkin (KMO) measure of sampling adequacy to determine the underlying proportion explained by individual items (6). Items with a KMO value <0.5 were iteratively removed, starting with the item with the lowest value until all individual remaining variables had a KMO value >0.5 (6). Analyses were performed using the remaining items. Since data were not normally distributed we choose principal axis factoring as factor extraction method (7), and since data were ordinal we choose for polychoric correlation (8). We determined the number of factors in the EFA by performing parallel analysis (number of iterations: 1000).
We first attempted oblique rotation (“Oblimin”). If factor intercorrelations were low (all <0.32), we used orthogonal rotation (“Equamax”) (9).
Because of the low sample size: 1) only factors with at least four items loading >0.6 were viewed as reliable, 2) if this condition was satisfied all items loading >0.5 were used for factor naming (6).

*Assumption testing multiple linear regression models*Standardized residuals (mean = 0, SD = 1) were used to identify possible outliers (10). Influential points were identified using Cook’s distance, with the cutoff set at 4/(n of patients – n of variables – 1). Scores >1 were regarded as potentially impactful (11). If influential points or outliers were identified we performed sensitivity analyses (rerunning the model after removing the influential points). Multicollinearity was checked using variance inflation factor (VIF), with the cutoff set at (square root(VIF) ≤ 2) (11). Homoscedasticity, linearity and multivariate normality were visually assessed using scale-location plots, residuals versus fitted value plots and a Q-Q plot of the studentized residuals. Lastly, independence of errors was tested using the Durbin-Watson test.

*Calculated statistics multiple linear models*Next to regular beta coefficients, we calculated standardized beta coefficients for continuous variables to improve comparability. These can be interpreted as: how many SD will the dependent variable change per SD increase of the independent variable. Using bias corrected and accelerated ordinary non-parametric bootstrapping we calculated 95% confidence intervals of the R^2^ of our multiple regression models (package: boot (12)). We calculated the adjusted R^2^ (adjusting R^2^ for number of independent variables) using the Pratt formula, which performs well if sample size to independent variables ratio is low (13).

*Explorative models using glmulti (14)*Models were specified as linear regressions. With 16 included possible variables a total of 2^16^=65536 possible models were generated. Estimates were based on the ~10% (6500 models) with lowest Akaike information criterions. An average importance of 0.8 or higher was regarded as reliable concerning our sample size (14).

**References**

1. Clinical Practice Guideline for the Evaluation and Management of Chronic Kidney Disease. Kidney International Supplements. Kidney Disease: Improving Global Outcomes (KDIGO) CKD Work Group; 2013. p. 1-150.

2. Lang RM, Badano LP, Mor-Avi V, Afilalo J, Armstrong A, Ernande L, et al. Recommendations for Cardiac Chamber Quantification by Echocardiography in Adults: An Update from the American Society of Echocardiography and the European Association of Cardiovascular Imaging. Journal of the American Society of Echocardiography. 2015;28(1):1-39.e14.

3. Lang RM, Bierig M, Devereux RB, Flachskampf FA, Foster E, Pellikka PA, et al. Recommendations for chamber quantification: a report from the American Society of Echocardiography's Guidelines and Standards Committee and the Chamber Quantification Writing Group, developed in conjunction with the European Association of Echocardiography, a branch of the European Society of Cardiology. Journal of the American Society of Echocardiography : official publication of the American Society of Echocardiography. 2005;18(12):1440-63.

4. Petersen SE, Aung N, Sanghvi MM, Zemrak F, Fung K, Paiva JM, et al. Reference ranges for cardiac structure and function using cardiovascular magnetic resonance (CMR) in Caucasians from the UK Biobank population cohort. Journal of Cardiovascular Magnetic Resonance. 2017;19(1):18.

5. Revelle W. psych: Procedures for Personality and Psychological Research. 1.7.5 ed. Evanston, Illinois, USA: Northwestern University; 2017.

6. Field A. Exploratory factor analysis. Discovering Statistics using SPSS. 3rd ed: Sage Publications Ltd.; 2009. p. 627-85.

7. Costello AB, Osborne JW. Best Practices in Exploratory Factor Analysis: Four Recommendations for Getting the Most From Your Analysis. Practical Assessment, Research & Evaluation. 2005;10(7).

8. Holgado–Tello FP, Chacón–Moscoso S, Barbero–García I, Vila–Abad E. Polychoric versus Pearson correlations in exploratory and confirmatory factor analysis of ordinal variables. Quality & Quantity. 2008;44(1):153.

9. Brown JD. Choosing the Right Type of Rotation in PCA and EFA. Shiken: JALT Testing & Evaluation SIG Newsletter. 2009;13(3):20-5.

10. Field A. Regression. Discovering Statistics using SPSS. 3rd ed: Sage Publications Ltd.; 2009. p. 197-263.

11. Kabacoff RI. Regression. R in Action, Data anlysis and graphics with R: Manning; 2015. p. 167-211.

12. Canty A, Ripley R. boot: Bootstrap R (S-Plus) Functions. 1.3-20 ed2017.

13. Yin P, Fan X. Estimating R2; Shrinkage in Multiple Regression: A Comparison of Different Analytical Methods. The Journal of Experimental Education. 2001;69(2):203-24.

14. Calcagno V, de Mazancourt C. glmulti: An R Package for Easy Automated Model Selection with (Generalized) Linear Models. 2010. 2010;34(12):29.
