# Supplementary material for: Depressive symptoms in Fabry disease: the importance of coping, subjective health perception and pain
Source: Orphanet J Rare Dis. 2020 Jan 28;15:28. doi: 10.1186/s13023-020-1307-y (PMC6986064; doi:10.1186/s13023-020-1307-y)
Supplement: Supplementary file 2 — Additional file 2. Supplemental results [file 13023_2020_1307_MOESM2_ESM.docx]

**Supplemental results***Contents*

- Exploratory factor analysis of coping list
  - Factor naming
  - Supplemental table 1 Variance explained by three factor structure
  - Supplemental table 2 Three factor structure matrix
- Assumption testing multiple linear regression models

*Exploratory factor analysis of coping list*Two patients did not fully complete the Utrecht Coping List (both missing one item). These two items were assumed to be missing completely at random and were imputed using the median of the answers of all other patients. The Kaiser-Meyer-Olkin (KMO) measure of sampling adequacy was 0.58, showing mediocre proportion of variance explained by underlying factors (1). We iteratively removed six items with an item KMO <0.5 (items 4, 9, 14, 27, 30, 40), which improved the overall KMO to 0.66. Following analyses were performed with the remaining 41 items. Using parallel analysis we determined that eigenvalues of four factors were higher compared to sampled data (**Supplemental figure 1**) (2).


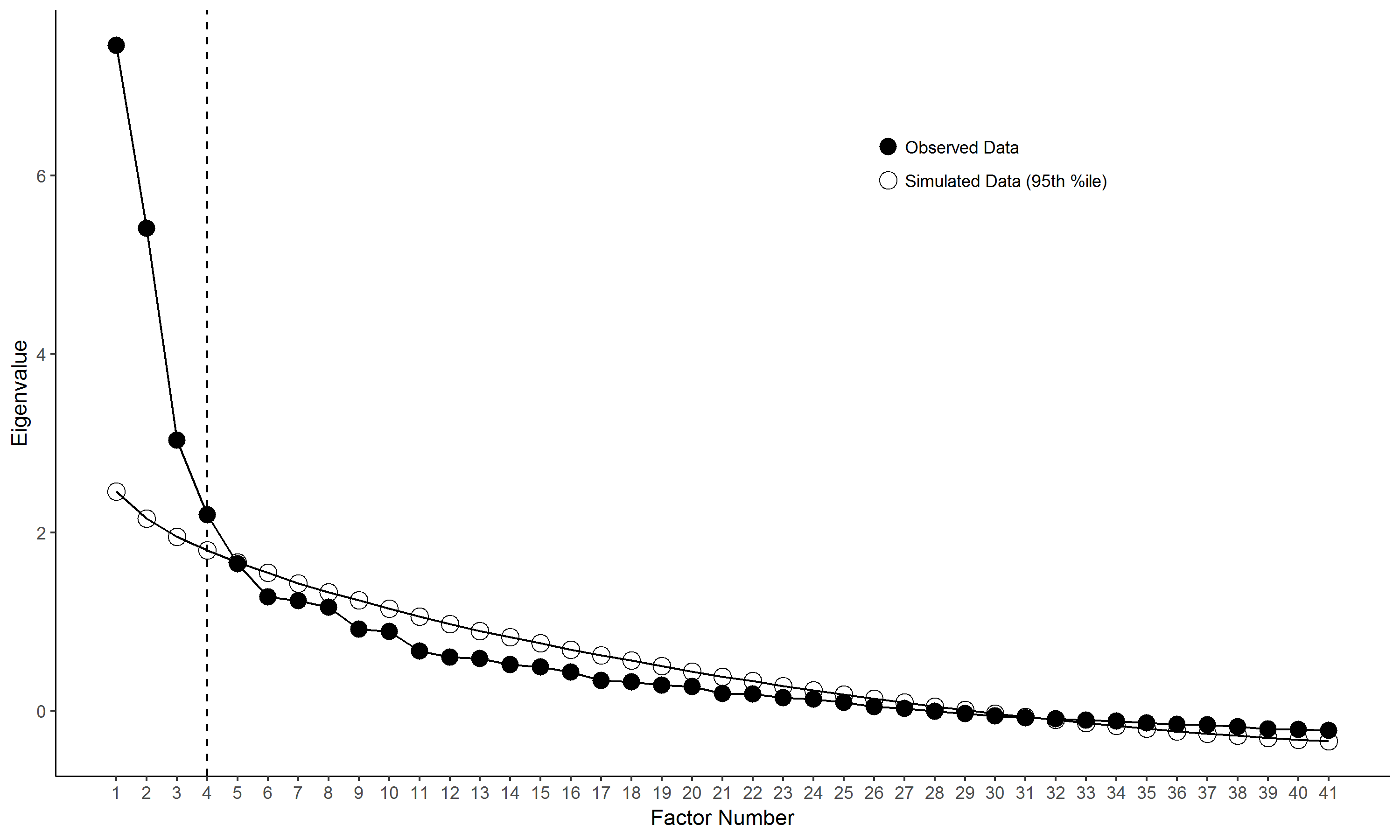


**Supplemental figure 1.** *Eigenvalues per factor of study dataset versus simulated dataset*

After oblique rotation factor intercorrelations were low (all <0.32) (3). Therefore we used orthogonal rotation (“Equamax”) for the final model.
The four extracted factors explained 39.9% of variance. Since the fourth factor consisted of only one item loading >0.5, this factor was regarded as unreliable and discarded. Therefore, we also analyzed a three factor structure which explained 35.5% of variance (**Supplemental table 1**), showed comparable fit and more simplicity compared to the four factor structure and was therefore preferred. All three factors showed ≥4 items loading >0.6 and a total of 25 out of 41 variables loaded >0.5 (**Supplemental table 2**).

*Factor naming*

The first factor consisted of ten items loading >0.5. Four items were originally included in the “passive” coping style (items 3, 24, 31, 46), three items were originally included in the “avoidance” coping style (items 8, 19, 26) and two items were originally included in the “palliative” coping style (items 6, 34) and one item (items 28) was originally not included in any of the predefined coping styles (4). The latter item was: in case of problems or unpleasant events how often do you “wait for better times”. Recurring themes in the items in this factor were: “avoiding or evading problems”, “seeking distraction to not think about the problem”, “self-isolation” and “brooding on problems”. We interpreted this as a combination of avoiding (thinking about) problems, partly by seeking (mental) distraction, but not being able to distract oneself completely resulting in brooding. We will refer to this factor as the “*avoiding and brooding*” coping style.

The second factor consisted of nine items loading >0.5. Six items were originally included the “active” coping style (items 13, 18, 21, 22, 23, 32). Two items were originally not included in any of the predefined coping styles. These two items were: in case of problems or unpleasant events how often do you “remain optimistic about the future” (item 20) and “see the humorous side of problems” (item 41). One item was originally included in the “Avoidance” coping scale. The item was: in case of problems or unpleasant events how often do you think “don’t worry: everything will be fine” (item 45). Recurring themes in the items of this factor were: “remaining calm and positive”, “using humor”, “see problems as challenges” and “analyze problems and seek solutions”. We interpreted this as having a positive attitude towards problems and seeing them as challenges to overcome. We will refer to this factor as the *“positivity and problem solving”* coping style.

The third factor consisted of six items loading >0.5. Five items were originally included in the “seeking social support” coping style (items 10, 38, 39, 42, 43). One item was originally included in the “reassuring thoughts” coping style (item 47). The item was: in case of problems or unpleasant events, how often do you “encourage yourself”. Recurring themes in the items of this factor were: “sharing feelings and doubts”, “discussing problems” and “seeking encouragement and understanding”. We will refer to this factor as the *“seeking social support and comfort”* coping style.

| **Supplemental table 1** Variance explained by three factor structure | | | |
| --- | --- | --- | --- |
|  | **Factor 1** | **Factor 2** | **Factor 3** |
| Proportion Variance, % | 13.8 | 11.5 | 10.3 |
| Cumulative Variance, % | 13.8 | 25.2 | 35.5 |

| **Supplemental table 2** Three factor structure matrix | | | |
| --- | --- | --- | --- |
| **Questions** | **Factor 1** | **Factor 2** | **Factor 3** |
| UCL1 | 0,376 | 0,169 | 0,168 |
| UCL2 | 0,070 | 0,305 | 0,210 |
| UCL3 | **0,696** | -0,062 | -0,048 |
| UCL5 | 0,479 | -0,360 | 0,046 |
| UCL6 | **0,569** | -0,251 | -0,036 |
| UCL7 | 0,170 | 0,055 | 0,267 |
| UCL8 | **0,583** | -0,075 | 0,141 |
| UCL10 | -0,029 | 0,023 | **0,723** |
| UCL11 | -0,209 | 0,383 | 0,266 |
| UCL12 | 0,271 | 0,373 | 0,045 |
| UCL13 | -0,105 | **0,528** | -0,018 |
| UCL15 | 0,359 | -0,122 | -0,072 |
| UCL16 | 0,380 | -0,099 | 0,102 |
| UCL17 | 0,373 | 0,044 | 0,310 |
| UCL18 | -0,029 | **0,649** | 0,275 |
| UCL19 | **0,609** | -0,262 | -0,042 |
| UCL20 | -0,366 | **0,610** | -0,008 |
| UCL21 | -0,238 | **0,540** | -0,097 |
| UCL22 | -0,290 | **0,647** | 0,212 |
| UCL23 | -0,241 | **0,683** | 0,162 |
| UCL24 | **0,531** | -0,252 | 0,035 |
| UCL25 | 0,150 | 0,061 | 0,337 |
| UCL26 | **0,615** | -0,239 | -0,129 |
| UCL28 | **0,690** | -0,046 | 0,031 |
| UCL29 | -0,048 | 0,261 | 0,452 |
| UCL31 | **0,638** | -0,068 | 0,031 |
| UCL32 | -0,082 | **0,669** | 0,119 |
| UCL33 | 0,164 | -0,080 | 0,214 |
| UCL34 | **0,537** | 0,123 | 0,190 |
| UCL35 | 0,363 | 0,209 | 0,370 |
| UCL36 | 0,337 | 0,193 | 0,137 |
| UCL37 | 0,194 | 0,484 | 0,137 |
| UCL38 | -0,082 | 0,198 | **0,667** |
| UCL39 | 0,098 | 0,022 | **0,798** |
| UCL41 | -0,047 | **0,574** | -0,041 |
| UCL42 | 0,030 | 0,045 | **0,717** |
| UCL43 | 0,010 | 0,091 | **0,604** |
| UCL44 | 0,439 | -0,040 | -0,483 |
| UCL45 | -0,003 | **0,504** | -0,307 |
| UCL46 | **0,630** | -0,107 | 0,053 |
| UCL47 | 0,251 | 0,342 | **0,534** |
| *UCL = Utrecht Coping List* | | | |

*Assumption testing multiple linear regression models*Model 1: Sensitivity analyses removing the most influential points did not improve the R^2^ and showed little effect on beta’s of the included variables. Other assumptions were assessed as described in the extensive methodology and were met.

Model 2: One potential outlier was also marked as most influential using Cook’s distance. Sensitivity analyses removing this point improved the R^2^ with ~2.5%. This patient scored highest on the depression questionnaire and we regarded this as representative of the extreme end of depressive symptoms in Fabry disease. Therefore, we choose to present model 2 with this patient included. However, using model 2 a slight underestimation of depression scores might occur at the extreme end. Other assumptions were met. **References**

1. Field A. Exploratory factor analysis. Discovering Statistics using SPSS. 3rd ed: Sage Publications Ltd.; 2009. p. 627-85.

2. Sakaluk JK, Short SD. A Methodological Review of Exploratory Factor Analysis in Sexuality Research: Used Practices, Best Practices, and Data Analysis Resources. Journal of sex research. 2017;54(1):1-9.

3. Brown JD. Choosing the Right Type of Rotation in PCA and EFA. Shiken: JALT Testing & Evaluation SIG Newsletter. 2009;13(3):20-5.

4. Schreurs PJ, Willige GV, Brosschot JF, Tellegen B, Graus GMH. Handleiding Utrechtse Coping Lijst UCL (herziene versie) [Instruction manual Utrecht Coping List UCL (revised version)]. Lisse: Swets & Zeitlinger; 1993.
