# Supplementary material for: Depressive symptoms in Fabry disease: the importance of coping, subjective health perception and pain
Source: Orphanet J Rare Dis. 2020 Jan 28;15:28. doi: 10.1186/s13023-020-1307-y (PMC6986064; doi:10.1186/s13023-020-1307-y)
Supplement: Supplementary file 3 — Additional file 3. Supplemental identified variables [file 13023_2020_1307_MOESM3_ESM.docx]

**Supplemental identified variables: Depressive symptoms in FD literature and variables of interest***Content*

- Search for studies on variables related to depressive symptoms in FD
- Identified studies on variables related to depressive symptoms in Fabry disease
- Supplemental table 3 Assessed variables related to depressive symptoms in Fabry disease literature per study
- Supplemental table 4 Summary variables related to depressive symptoms in Fabry disease literature
- Supplemental table 5 Variables significantly related to depressive symptoms in the general population and unexplored in Fabry disease literature

*Search for studies on variables related to depressive symptoms in FD*
In addition to the studies extracted from the systematic review (1), we searched Pubmed for studies on depressive symptoms in FD published until the 7^th^ of January 2019 using the following criteria:

| *Depressive symptoms:* ((((("Neuropsychiatry"[Mesh]) OR Neuropsychiatr*)) OR ((depressive disorder) OR (((((depression) OR ("Depression"[Mesh] AND "Depressive Disorder"[Mesh])) OR depressive symptoms) OR psychology) OR psychiatry)))) |
| --- |
| *Fabry disease:*  AND  (fabry*[tiab]) OR (alpha galactosidase a deficien*[tiab]) OR (angiokeratoma corporis diffusum[tiab]) |

*Identified studies on variables related to depressive symptoms in Fabry disease
Included studies*
In total, we identified 16 studies assessing the relation between variables and depressive symptoms in Fabry disease. Seven studies by using the systematic review (2-8), eight studies using the Pubmed search (9-16) and one book chapter presenting new data by screening the reference lists of included studies (17).

*Excluded studies*Five studies in the systematic review were excluded since no relations were tested between variables to depressive symptoms in FD (18-22).
Of the 104 studies from the Pubmed search, 92 were excluded after screening of title and abstract. Four more studies were excluded after full text evaluation (no relation tested between variables and depressive symptoms (23, 24), included only pediatric patients (25, 26)).

| **Supplemental table 3** Assessed variables related to depressive symptoms in Fabry disease literature per study (alphabetic order) | | | | | | | |
| --- | --- | --- | --- | --- | --- | --- | --- |
| **First author** | **Year** | **Patients, n (men)** | **Age (years), median or mean ±SD (range)** | **Study design** | **Measure of depressive symptoms** | **Variables significantly related to depressive symptoms in FD** | **Variables not significantly related to depressive symptoms in FD** |
| Ali (12) | 2017 | 10 (2) | 42.1±12.0 (22-61) | Prospective, longitudinal | ASEBA ASR OASR | (Tele)counseling, change over time in SF-36 mental health, change over time in BPI severity | Change over time in adaptive functioning, change over time in SF-36 physical health, change over time in BPI interference with life |
| Cole (2) | 2007 | 184 (74) | 44±14 (18–76) | Prospective, cross-sectional | CESD | Other chronic illness present, interference of FD symptoms with life, acroparesthesia, anhidrosis, unemployement, abdominal symptoms, cardiac symptoms, ERT, No partner^&^, Problems with income^&^ | Age, sex, level of education, having child(ren) with FD, cerebrovascular symptoms, renal symptoms, time on ERT, response of symptoms to ERT |
| Crosbie (3) | 2009 | 28 (16) | (18-60) | Prospective, cross-sectional | MMPI-2 depression subscale | Experienced symptom severity, pain severity | Time on ERT, time since diagnosis |
| Franzen (14) | 2015 | 52 (17) | 42.8±14.7 | Prospective, cross-sectional | PHQ-9 | Epworth sleepiness scale | - |
| Grewal (4) | 1993 | 33 (6) | 29.3 (23-37)^†^ | Retrospective, longitudinal | Reported diagnosis of depression | Acroparesthesia and pain crises | - |
| **Supplemental table 3** Assessed variables related to depressive symptoms in Fabry disease literature per study (alphabetic order), continued | | | | | | | |
| **Author** | **Year** | **Patients, n (men)** | **Age (years), median or mean ±SD (range)** | **Study design** | **Measure of depressive symptoms** | **Variables significantly related to depressive symptoms in FD** | **Variables not significantly related to depressive symptoms in FD** |
| Körver (16) | 2018 | 81 (28) | 44.5±14.3 | Prospective, cross-sectional | CESD | Subjective cognitive complaints | Objective cognitive impairment, sex and phenotype |
| Laaksonen (5) | 2008 | 12 (0) | 45.5±15.1 (17-63) | Prospective, cross-sectional | GCPS depression questions | Age, experienced somatic symptoms, neuropathic pain, decreased IENFD | - |
| Laney (6) | 2010 | 33 (15) | 40 (18-59) | Prospective, cross-sectional | ASR and ABCL, DSM depression scale | Poor adaptive functioning | - |
| Lelieveld (11)^‡^ | 2015 | 14 (4) | 46.1±10.8 (27-64) | Prospective, longitudinal | HAMD-17 | - | Age, neuropsychological measures, brain structural parameters |
| Loeb (13) | 2018 | 41 (12) | 47.2±14.7 (20-75) | Prospective, cross-sectional | HAMD-17 | Subjective cognitive complaints | Sex, objective cognitive impairment |
| Löhle (10) | 2015 | 110 (50) | 49.0±16.0 (17-84) | Prospective, cross-sectional | BDI-II | MSSI total | - |
| Müller (17) | 2006 | 36 (18) | 36±10 | Prospective, cross-sectional | HAMD, reported diagnosis of depression | Social support | - |
| Schermuly (7) | 2011 | 25 (10) | 36.5±11.0 (21-56) | Prospective, cross-sectional | HAMD-17 | PANSS positive symptoms, PANSS negative symptoms, SF-36 mental health | Cognitive performance, SF-36 physical health, BPI severity, BPI interference with life, age, WMLL |
| **Supplemental table 3** Assessed variables related to depressive symptoms in Fabry disease literature per study (alphabetic order), continued | | | | | | | |
| **Author** | **Year** | **Patients, n (men)** | **Age (years), median or mean ±SD (range)** | **Study design** | **Measure of depressive symptoms** | **Variables significantly related to depressive symptoms in FD** | **Variables not significantly related to depressive symptoms in FD** |
| Segal (8) | 2010 | 16 (7) | 29 (7-61) | Prospective, cross-sectional | DSM-IV criteria | Overall FD involvement, decreased cognitive function | - |
| Sigmundsdottir (9) | 2014 | 17 (12) | 46.6±11.8 (25-60) | Prospective, cross-sectional | DASS-21 depression subscale | MSSI neurologic, TIA/stroke, BPI severity, BPI intensity | Age, MSSI total, MSSI general, MSSI cardiac, MSSI renal, CKD |
| Talbot (15) | 2016 | 20 (20) | 43.9±10.7 (23-71) | Prospective, cross-sectional | Clinical diagnosis (symptoms of depression, treatment) | Periodic limb movement index | - |
| *ABCL = Achenbach adult behavior checklist, ASEBA = Achenbach System of Empirically Based Assessment, ASR = Adult Self-Report, BDI-II = Beck Depression Inventory II, BPI = brief pain inventory, CESD = The Centre for Epidemiological Studies Depression scale, CKD = chronic kidney disease (stages according to KDIGO guidelines), Cross = cross-sectional, DASS-21 = Depression, Anxiety and Stress scale, DSM = Diagnostic and Statistical Manual of Mental Disorders, ERT = enzyme replacement therapy, FD = Fabry disease, GCPS = modified graded chronic pain status questionnaire, HAMD = Hamilton Rating Scale for Depression, HAMD-17 = HAMD 17-item version, IENFD = intraepidermal nerve fiber density, long = longitudinal, MMPI-2 = Minnesota Muliphasic Personality Inventory, MSSI = Mainz Severity Score Index, OASR = Older Adult Self Report, PANSS = The Positive and Negative Syndrome Scale, PHQ-9 = patient health questionnaire-9, pros = prospective, retro = retrospective, SF-36 = 36-Item Short Form Survey, WMLL = white matter lesion load*  *& Relation was claimed, however calculated odds ratios crossed 1. † Age at diagnosis. – Not available. ‡ This study is an eight year follow-up study of a subgroup of the study by Schermuly, Muller (7).* | | | | | | | |

| **Supplemental table 4** Summary variables related to depressive symptoms in Fabry disease literature, use in models en description | | | | |
| --- | --- | --- | --- | --- |
| **Variables assessed in relation to depressive symptoms** | **Significant relation found between variable and depressive symptoms [references]** | **Included in model 1 and 2** | **Included in explorative models** | **Name variable assessed in relation to depressive symptoms current study** |
| *Age, sex and phenotype* | | | | |
| Age | No (2, 7, 9, 11), Yes (5) | No | No | NA |
| Sex | No (2, 13) | No | No | NA |
| Sex and phenotype | No (16) | No | No | NA |
| *Subjective/reported symptoms* | | | | |
| Overall experienced symptoms | Yes (2, 3, 5) | Yes | Yes | SF-36 general health perception |
| Abdominal, renal, cerebrovascular, cardiac symptoms | Yes (2) | No | No | NA |
| Subjective cognitive complaints | Yes (13, 16) | No | Yes | Subjective cognitive complaints |
| *Symptoms, organ involvement, disease severity* | | | | |
| Cerebral: |  |  |  |  |
| TIA/stroke | Yes (9) | Yes | Yes | History of stroke |
| WMLs | No (7) | No | Yes | Fazekas scale |
| Brain structural parameters | No (11) | No | No | NA |
| Cognitive functioning | No (7, 11, 13, 16), Yes (8) | No | No | NA |
| MSSI neurologic | Yes (9) | No | No | NA |
| Clinical involvement: |  |  |  |  |
| Overall | Yes (8) | No | No | NA |
| Anhidrosis | Yes (2) | No | No | NA |
| **Supplemental table 4** Summary variables related to depressive symptoms in Fabry disease literature, use in models en description (continued) | | | | |
| **Variables assessed in relation to depressive symptoms** | **Significant relation found between variable and depressive symptoms [references]** | **Included in model 1 and 2** | **Included in explorative models** | **Name variable assessed in relation to depressive symptoms current study** |
| CKD | No (9) | No | Yes | Renal and/or cardiac involvement (see supplemental table 6 below) |
| MSSI total | No (9), Yes (10) | No | No | NA |
| MSSI general, cardiac, renal | No (9) | No | No | NA |
| Pain: |  |  |  |  |
| Neuropathic pain (acroparesthesia) | Yes (2, 4, 5) | No | No | NA |
| Decreased IENFD | Yes (5) | No | No | NA |
| BPI subscales | No (7, 12), Yes (3, 9, 12) | Yes | Yes | BPI severity scale |
| Sleep: |  |  |  |  |
| Periodic limb movement index | Yes (15) | No | No | NA |
| Excessive daytime sleepiness | Yes (14) | No | Yes | PSQI |
| *Treatment* |  |  |  |  |
| (Tele)counseling | Yes (12) | No | No | NA |
| ERT use and subjective response | No (2) | No | No | NA |
| Time on ERT | No (2, 3) | No | No | NA |
| *Background and patient characteristics* |  |  |  |  |
| Patient characteristics: |  |  |  |  |
| Time since diagnosis | No (3) | No | No | NA |
| **Supplemental table 4** Summary variables related to depressive symptoms in Fabry disease literature, use in models en description (continued) | | | | |
| **Variables assessed in relation to depressive symptoms** | **Significant relation found between variable and depressive symptoms [references]** | **Included in model 1 and 2** | **Included in explorative models** | **Name variable assessed in relation to depressive symptoms current study** |
| Other chronic illness | Yes (2) | Yes | Yes | Comorbidity |
| Adaptive functioning | No (12), Yes (6) | No | No | NA |
| Background: |  |  |  |  |
| No partner | Unclear (2) | Yes | Yes | Relationship status (Partner/Single) |
| Problems with income | Unclear (2) | No | No | NA |
| Level of education | No (2) | No | No | NA |
| Unemployment | Yes (2) | Yes | Yes | Unfit for work |
| Child(ren) with Fabry disease | No (2) | No | No | NA |
| Social support | Yes (17) | No | Yes | SF-36 Social functioning |
| *Other* |  |  |  |  |
| SF-36 mental health | Yes (7, 12) | No | No | NA |
| SF-36 physical health | No (7, 12) | No | No | NA |
| PANSS positive symptoms | Yes (7) | No | No | NA |
| PANSS negative symptoms | Yes (7) | No | No | NA |
| *BPI = brief pain inventory, CKD = chronic kidney disease, ERT = enzyme replacement therapy, IENFD = intraepidermal nerve fiber density, MSSI = Mainz severity score index, NA = Not assessed, PANSS = positive and negative syndrome scale, SF-36 = 36-Item Short Form Survey, WMLs = white matter lesions* | | | | |

| **Supplemental table 5** Variables significantly related to depressive symptoms in the general population and unexplored in Fabry disease literature, use in models en description | | | | |
| --- | --- | --- | --- | --- |
| **Variables assessed in relation to depressive symptoms** | **Scientific foundation** | **Name variable assessed in relation to depressive symptoms current study (variable description and coding)** | **Included model 2** | **Included in explorative models** |
| Coping | MA: Coping styles are positively and negatively related to depressive symptoms (27). | “Avoidance and brooding”, “positivity and problem solving” and “seeking social support and comfort” | Yes | Yes |
| Renal and/or cardiac involvement | MA&R: chronic kidney disease, dialysis and kidney transplantation are associated with depression (28, 29).  MA & R: depression is a risk factor for cardiac disease (30) and occurs more often in patients with cardiac disease (31). | Renal and/or cardiac involvement (No renal or cardiac involvement (0), Renal involvement (eGFR <60ml/min/1.73m^2^) and/or cardiac involvement (left ventricular hypertrophy or fibrosis) (1), Cardiac and/or renal complications (2)) | No | Yes |
| History of depression | R: History of depression increases risk of recurrent depression (32, 33). | History of depression (Absent (0)/Present (1)) | No | Yes |
| Loneliness | MA: Loneliness is associated with depression (34). | Loneliness (Absent (0)/Present (1)) | No | Yes |
| Fatigue | SR: Fatigue associated with depression in cancer patients (35) and rheumatoid arthritis patients (36). | SF-36 Fatigue (scale, possible range 0-100) | No | Yes |
| *CKD = chronic kidney disease, EFA = exploratory factor analysis, eGFR = estimated glomerular filtration rate, LVH = left ventricular hypertrophy, MA = Meta-analysis, PSQI = Pittsburgh sleep quality index, R = review, SF-36 = 36-Item Short Form Survey, SR = systematic review* | | | | |

**References**

1. Bolsover FE, Murphy E, Cipolotti L, Werring DJ, Lachmann RH. Cognitive dysfunction and depression in Fabry disease: a systematic review. J Inherit Metab Dis. 2014;37(2):177-87.

2. Cole AL, Lee PJ, Hughes DA, Deegan PB, Waldek S, Lachmann RH. Depression in adults with Fabry disease: a common and under-diagnosed problem. J Inherit Metab Dis. 2007;30(6):943-51.

3. Crosbie TW, Packman W, Packman S. Psychological aspects of patients with Fabry disease. Journal of Inherited Metabolic Disease. 2009;32(6):745-53.

4. Grewal RP. Psychiatric disorders in patients with Fabry's disease. International journal of psychiatry in medicine. 1993;23(3):307-12.

5. Laaksonen SM, Roytta M, Jaaskelainen SK, Kantola I, Penttinen M, Falck B. Neuropathic symptoms and findings in women with Fabry disease. Clinical neurophysiology : official journal of the International Federation of Clinical Neurophysiology. 2008;119(6):1365-72.

6. Laney DA, Gruskin DJ, Fernhoff PM, Cubells JF, Ousley OY, Hipp H, et al. Social-adaptive and psychological functioning of patients affected by Fabry disease. J Inherit Metab Dis. 2010;33 Suppl 3:S73-81.

7. Schermuly I, Muller MJ, Muller KM, Albrecht J, Keller I, Yakushev I, et al. Neuropsychiatric symptoms and brain structural alterations in Fabry disease. European journal of neurology. 2011;18(2):347-53.

8. Segal P, Kohn Y, Pollak Y, Altarescu G, Galili-Weisstub E, Raas-Rothschild A. Psychiatric and cognitive profile in Anderson-Fabry patients: a preliminary study. J Inherit Metab Dis. 2010;33(4):429-36.

9. Sigmundsdottir L, Tchan MC, Knopman AA, Menzies GC, Batchelor J, Sillence DO. Cognitive and psychological functioning in Fabry disease. Archives of clinical neuropsychology : the official journal of the National Academy of Neuropsychologists. 2014;29(7):642-50.

10. Lohle M, Hughes D, Milligan A, Richfield L, Reichmann H, Mehta A, et al. Clinical prodromes of neurodegeneration in Anderson-Fabry disease. Neurology. 2015;84(14):1454-64.

11. Lelieveld IM, Bottcher A, Hennermann JB, Beck M, Fellgiebel A. Eight-Year Follow-Up of Neuropsychiatric Symptoms and Brain Structural Changes in Fabry Disease. PloS one. 2015;10(9):e0137603.

12. Ali N, Gillespie S, Laney D. Treatment of Depression in Adults with Fabry Disease. JIMD reports. 2017;38:13-21.

13. Loeb J, Feldt-Rasmussen U, Madsen CV, Vogel A. Cognitive Impairments and Subjective Cognitive Complaints in Fabry Disease: A Nationwide Study and Review of the Literature. JIMD reports. 2018;41:73-80.

14. Franzen D, Gerard N, Bratton DJ, Wons A, Gaisl T, Sievi NA, et al. Prevalence and Risk Factors of Sleep Disordered Breathing in Fabry disease: A Prospective Cohort Study. Medicine. 2015;94(52):e2413-e.

15. Talbot A, Hammerschlag G, Goldin J, Nicholls K. Sleep Disturbance, Obstructive Sleep Apnoea and Abnormal Periodic Leg Movements: Very Common Problems in Fabry Disease. JIMD reports. 2016;31:37-44.

16. Körver S, Geurtsen GJ, Hollak CEM, van Schaik IN, Longo MGF, Lima MR, et al. Predictors of objective cognitive impairment and subjective cognitive complaints in patients with Fabry disease. Scientific Reports. 2019;9(1):188.

17. Müller MJ. Neuropsychiatric and psychosocial aspects of Fabry disease. In: Mehta A, Beck M, Sunder-Plassmann G, editors. Fabry Disease: Perspectives from 5 Years of FOS. Oxford: Oxford PharmaGenesis; 2006.

18. Wang RY, Lelis A, Mirocha J, Wilcox WR. Heterozygous Fabry women are not just carriers, but have a significant burden of disease and impaired quality of life. Genetics in medicine : official journal of the American College of Medical Genetics. 2007;9(1):34-45.

19. Miners AH, Holmes A, Sherr L, Jenkinson C, MacDermot KD. Assessment of health-related quality-of-life in males with Anderson Fabry Disease before therapeutic intervention. Quality of life research : an international journal of quality of life aspects of treatment, care and rehabilitation. 2002;11(2):127-33.

20. MacDermot KD, Holmes A, Miners AH. Anderson-Fabry disease: clinical manifestations and impact of disease in a cohort of 60 obligate carrier females. Journal of medical genetics. 2001;38(11):769-75.

21. MacDermot KD, Holmes A, Miners AH. Anderson-Fabry disease: clinical manifestations and impact of disease in a cohort of 98 hemizygous males. J Med Genet. 2001;38(11):750-60.

22. Sadek J, Shellhaas R, Camfield CS, Camfield PR, Burley J. Psychiatric findings in four female carriers of Fabry disease. Psychiatric genetics. 2004;14(4):199-201.

23. von der Lippe C, Frich JC, Harris A, Solbraekke KN. Experiences of Being Heterozygous for Fabry Disease: a Qualitative Study. Journal of genetic counseling. 2016;25(5):1085-92.

24. Sawada J, Katayama T, Kano K, Asanome A, Takahashi K, Saito T, et al. A Sporadic Case of Fabry Disease Involving Repeated Fever, Psychiatric Symptoms, Headache, and Ischemic Stroke in an Adult Japanese Woman. Internal medicine (Tokyo, Japan). 2015;54(23):3069-74.

25. Bugescu N, Naylor PE, Hudson K, Aoki CD, Cordova MJ, Packman W. The Psychosocial Impact of Fabry Disease on Pediatric Patients. Journal of Pediatric Genetics. 2016;5(3):141-9.

26. Bugescu N, Alioto A, Segal S, Cordova M, Packman W. The neurocognitive impact of Fabry disease on pediatric patients. American journal of medical genetics Part B, Neuropsychiatric genetics : the official publication of the International Society of Psychiatric Genetics. 2015;168b(3):204-10.

27. Penley JA, Tomaka J, Wiebe JS. The Association of Coping to Physical and Psychological Health Outcomes: A Meta-Analytic Review. Journal of Behavioral Medicine. 2002;25(6):551-603.

28. Susan Hedayati S, Yalamanchili V, Finkelstein FO. A practical approach to the treatment of depression in patients with chronic kidney disease and end-stage renal disease. Kidney International. 2012;81(3):247-55.

29. Palmer S, Vecchio M, Craig JC, Tonelli M, Johnson DW, Nicolucci A, et al. Prevalence of depression in chronic kidney disease: systematic review and meta-analysis of observational studies. Kidney International. 2013;84(1):179-91.

30. Van der Kooy K, van Hout H, Marwijk H, Marten H, Stehouwer C, Beekman A. Depression and the risk for cardiovascular diseases: systematic review and meta analysis. International journal of geriatric psychiatry. 2007;22(7):613-26.

31. Lespérance F, Frasure-Smith N. Depression in patients with cardiac disease: a practical review. Journal of Psychosomatic Research. 2000;48(4):379-91.

32. Belsher G, Costello CG. Relapse after recovery from unipolar depression: a critical review. Psychological bulletin. 1988;104(1):84-96.

33. Post RM. Transduction of psychosocial stress into the neurobiology of recurrent affective disorder. The American journal of psychiatry. 1992;149(8):999-1010.

34. Erzen E, Cikrikci O. The effect of loneliness on depression: A meta-analysis. The International journal of social psychiatry. 2018;64(5):427-35.

35. Brown LF, Kroenke K. Cancer-Related Fatigue and Its Associations With Depression and Anxiety: A Systematic Review. Psychosomatics. 2009;50(5):440-7.

36. Nikolaus S, Bode C, Taal E, van de Laar MA. Fatigue and factors related to fatigue in rheumatoid arthritis: a systematic review. Arthritis care & research. 2013;65(7):1128-46.
